# A Refugee Rose of competencies and capabilities for mental healthcare of refugees

**DOI:** 10.1192/bjo.2022.11

**Published:** 2022-02-14

**Authors:** Kamaldeep Bhui

**Affiliations:** Department of Psychiatry and Nuffield Department of Primary Care Health Sciences, University of Oxford, UK; East London NHS Foundation Trust, UK; Oxford Health NHS Foundation Trust, UK; and World Psychiatric Association Collaborating Centre in Research, Training, Policy and Practice, UK

**Keywords:** Refugee, mental health, culture, ethnography, eco-social

## Abstract

In this paper, I set out the challenges of care for refugees and suggest approaches to assessment and intervention. I discuss clinical interventions that can address the immediate concern of the clinician in a bio-psycho-social framework, and the value of considering eco-social and structural influences that can hinder recovery and perpetuate inequalities. Refugees face multiple adversities before, during and after escaping from life-threatening situations, political violence, torture and persecution. They present with complex health needs and encounter hostility from host countries and public services, which see their needs as an additional demand on the public purse. Regrettably, existing care practice and training of professionals do not often include skills for working across cultures, including cultural formulations and fair assessment, cultural adaptation of interventions, cultural competence and cultural consultation methods, including clinical ethnography and exploration of cultural identity and explanatory models. There are little data on effective and kind models of interpretation and translation. Care systems are rarely designed to fully address the needs of refugees. Health practitioners are not trained to address structural and institutional racism and discrimination, which leads to exclusion of the most marginalised, with little attention to social justice and fair processes as part of appropriate healthcare.

The United Nations Humanitarian Commission for Refugees (UNHCR) report^1^ of 2019 sets out the numbers of displaced persons globally, including refugees. There are 86.5 million people of concern to UNHCR, of which 4.1 million are asylum seekers, 20.4 million are refugees, 4.2 million are stateless persons, 5.7 million are returnees (refugees and internally displaced persons) and 43.5 million are internally displaced persons. Fifty-seven per cent of refugees come from Syria, Afghanistan and South Sudan. Thus, asylum seekers and refugees emerge from many distinct social and political contexts, from situations with decades and sometimes centuries of conflict affecting multiple generations. Sometimes refugees come from places facing famine, war or natural disasters; there are variable time periods between experienced persecution, torture or threat to life and the decision to flee; and then the duration of stay in the host country, and support and adversity during this period, will also shape the mental health consequences of earlier trauma and help-seeking.^2^ The premigration context from which refugees flee can also vary from relatively stable and resourced health and social systems with relatively safe and politically stable situations, to countries with extreme poverty and widespread health-related problems, all compounded by weak governance in public systems.

The humanitarian crisis facing refugees can be compounded by legal action in host countries that leads to precarity, marginalisation, criminalisation and victimisation.^3^ The diverse journeys people take to escape untenable situations in their countries of origin are themselves a threat to their lives; for example, hazardous sea crossings, paying intermediaries to escape and being criminalised, enslaved or subject to exploitation or sexual abuse.^4–6^ Children and unaccompanied minors are especially at risk of such hazards. Given the life-course risks of poor health (physical and mental), more life years lost and years of life lived with disability among people experiencing trauma and forced displacement, preventive interventions for young people might provide the greatest lifetime benefits. Thus, the response required is a multisystem bioecological set of actions, at individual, familial, community, school, institutional and policy levels, to benefit mental health and psychosocial well-being of refugee children.^7^ The greatest impacts are secured by multilevel interventions addressing interactions between ecological systems, proximal processes and the agency of the developing refugee child.

Refugees thus face multiple adversities and many potential journeys. There are marked variations in the post-migration experience in asylum countries, including social isolation, stigma, discrimination, gender violence, racism, criminalisation, unemployment and poverty.^8–11^ Any plans to respond to the health and social care needs of refugees must be cognisant of these multiple stories, from distinct countries, religious and cultural traditions, and the specific ecological and political niche in which their reason for fleeing evolved into a crisis. If people already suffer mental health problems at the time of fleeing, they will be more vulnerable to future mental health problems. Their ability to manage the stressors in their new environment with psychological flexibility may enable them to remain healthy and successful.^12^ Or, if their mental health is so compromised, they will struggle more than most if the support they experience does not nurture their talents and strengths. People of neurodiverse identities (autism, attention-deficit hyperactivity disorder, intellectual disability) experience adversity more intensely, and have greater difficulties overcoming the psychological and relational consequences, and therapeutic interventions need adaptation.^5^ Geographical mobility, housing instability and social isolation further compound poor health by exposure to more strains and hazards in the asylum-seeking journey.^13,14^

## Frameworks for assessment and intervention

These complexities mandate that assessments of mental health and care and treatment plans consider personal narratives and meaning-making, including attention to fractured moral frameworks, moral injury, social injustice and failures of state protections that are distressing, but not easily resolved through health systems. Furthermore, clinicians will need to attend to multiple social and cultural factors that influence the expression, management and meaning of evolving mental illness, as well as recovery from it. Specifically, people encountering mental health services should not be receiving care that is neglectful of their unique narratives and their location in intersectional positions, which are not anticipated by service designers and commissioners.

Refugees come from a range of national, cultural and religious contexts, requiring the clinician to engage with appropriate ways to assess psychopathology, and ascribe meaning to unusual or atypical symptoms of social or cultural distress that are easily pathologized and over-medicalised.^3^ Furthermore, from studies of cultural psychiatry, it is well-established that symptom expression varies by culture, and the interaction between doctor and patient influences the way people's distress is perceived, understood and responded to. As a consequence, the DSM-IV and DSM-5 recommend a cultural formulation interview, asking clinicians to pay attention to cultural identity and acculturation, explanatory models and health beliefs, cultural meanings and influences in the psychosocial environment, cultural perspectives on the clinician–patient relationship and an overall judgement on the impact of culture on diagnosis.^15^ The Cultural Formulation Interview in the DSM-5 gives more guidance on the areas of enquiry, yet the training emphasises narrative methods to ensure the patient's specific story is embraced, heard, noted and built upon,^16^ as set out the following quote:

‘ …. basic interviewing skills extend to the art of conducting a person-centered, culturally informed evaluation …. flexible responses to the specifics of the person, problem, setting, and other elements of the interview ….. shaped by the identity and social position of both patient and clinician.’^16^

These approaches have long been used in cultural psychiatry, yet never fully articulated and manualised for use in specific refugee populations. The DSM-IV and DSM-5 provide that opportunity, yet historically, the uptake is poor, given that biomedicine generally operates on universal principles assuming all peoples are physiologically and anatomically, and therefore socioculturally, sufficiently similar irrespective of cultural contexts. Health is affected not only by genes and biology, but also by the eco-social and psychosocial environment and social stressors over the life course. These adverse experiences (racism, poverty, isolation, unemployment) are manifestations of precarity, and drive poor health and inequalities.

Assessment and intervention for refugees must also embrace all we know from trauma studies about how adversity leads to poor health, weathering, inflammatory responses and shortened life expectancy. Medical liaison is therefore essential, requiring primary and specialist services to be integrated, and psychiatric and medical care to also be closely aligned. Adverse childhood experiences and traumatic experiences in adults both lead to physical and mental illnesses, and even shorter life expectancy.^17–21^ Thus, the clinical assessment, formulation, and care plan for asylum seekers and refugees has to reflect an understanding of the challenges faced by traumatised populations regarding post-traumatic symptoms and avoidance of reminders by the person suffering with poor health, as well as carers and service providers.

Refugees experience multiple social, political, ethical, legal and philosophical dilemmas when experiencing distress, and when they must make decisions about who can help them with the totality of their world view. Their perspectives cannot easily be mapped to discrete interventions that will be insensitive to wider aspects of their lived experience. The assessor and care system must also recognise historical antecedents to which the patient, or their parents, family and ancestors, may have been exposed; that is, the intergenerational transmission of trauma needs to be kept in mind. Although the bio-psycho-social approach is well established, there remain controversies in mental health settings about the balance of biological, social and psychological causes and related implications for therapeutics. Krieger proposes an eco-social approach that addresses these additional layers of history and contemporary adversity, as well as the biosocial interface.^6,22^ The assessor must be sensitive to the potential risk of multiple medical conditions and the way in which psychosocial adversity is embodied through physiological and psychological coping. Coping is not only about documenting discrete trauma events during the life course, but must be understood, and is conditioned by personal narratives and meanings assigned to the trauma and persecution, including historical narratives. More attention is needed around how structural sources of adversity, grounded in colonial histories, are sustained and affect marginalised people today.^3,7^

## Cultural competency

Many countries have embraced cultural competency as an essential requirement to work with culturally and ethnically diverse populations, irrespective of refugee status, yet we lack large trials and evidenced models.^23,24^ Most specialist services are found in high-income countries seeking to respond to the needs of migrants and their descendants. Most of these services are not sustained in public-funded programmes, often relying on the charity and voluntary sector to address shortfalls. Diagnostic frameworks must be suited to specific regions around the world, although the relevance and validity of the frameworks are challenged not only for cultural fit, but also for overly reifying specific ‘conditions’ that may themselves be culturally constructed.

In a National Health Service (NHS) Trust in East London, I evolved the cultural consultation model^25^ originally developed at McGill University,^26^ using aspects of the cultural formulation, incorporating the Barts Explanatory Model Interview and undertaking in-depth investigations of the narratives of both the referring clinicians, teams and organisations, as well as the patients. This approach showed that conflicts between patient and services were the product of a complex interplay of an ill-fitting service narratives and the actual patient experience. Although developed for a specific NHS context, within a UK-based system of commissioning and provision, the principles are relevant for all societies where cultural complexity may confound optimal assessment of mental health status and the allocation effective care pathways and interventions. There are core skills for clinicians that can be transported and adapted for local populations, but the services will be constrained by national political contexts and sentiments toward the framing of identity, ethnicity and race.^27^ The specific form in which cultural consultation is delivered varies between countries, and the local pathways and available interventions will also depend on resources from public services or from the charity sector. Thus, understanding the local assets and resources is a necessary part of offering such a service. Indeed, cultural competence and measures to improve services for migrants have been focused mostly in high-income countries, perhaps because of hostile attitudes toward migrants or the adoption of institutional frameworks for care provision (from the high-income countries) that do not fully attend to cultural diversity.^28^ The lack of resources devoted to mental health is part of the challenge in low- and middle-income countries, and so models of care will need to be realistic, sustainable and locally designed; for example, lay and peer workers can deliver psychological interventions with good effect, at much less cost.^29^ Clearly, this helps overcome language barriers to some extent, and reduces social isolation.^30^

Ethnography is part of the approach. This originates from anthropological research methods that were adapted into clinical practice. The value of this is that ethnography assumes nothing about the patient and locates the initial conversation as one of discovering the patient's world view, beliefs and positionality, rather than focusing on the often pressing administrative and clinical checklists. Furthermore, ethnography captures narratives of patients’ sources of suffering in an authentic manner, intact, and with all the complex interplays between intersectional webs of causations that often evade the biomedical and psychiatric gaze. The clinical task can over-prioritise the pressing pursuit of psychopathology and a formulation, drawing on familiar and trusted scripts to match – as quickly as possible – which therapeutic offers might be helpful. Thus clinical ethnography is a tool for assessment and understanding, and necessarily slows down this process to encourage careful reflection; it addresses not only the individual embodied experience of distress, but also the social nexus within which it arises, the beliefs and vocabularies of the patient and her kinship system, and the intersectional and social positionality of the person seeking help and the clinician.^31,32^ Ethnography and cultural consultation methods can also interrogate organisational practices that are institutionalised and unnoticed,^33,34^ yet these can give rise to the neglect of racism and the complex needs of marginalised groups; the risk is that such practices are silent on the coercive and criminalising responses from the state and institutions, when these interact with vulnerable populations.^33,35^ For example, if racism is part of the lived experience of those seeking help, and clinicians do not recognise it or ask about it, this reinforces a lack of connection with the patient and can lead to alienation and isolation. Similarly, if there are linguistic barriers, and the clinician is not skilled in working with interpreters, or the provider organisation or national policies do condone these or provide funds for such interpreting services,^36^ those with diverse linguistic needs will receive an inferior service. Communication is compromised, and the risks of miscommunication and mistakes in the classification of diagnostic labels and care pathways is inevitable and may lead to coercive care and unhelpful interventions with associated adverse consequences, and the patient may feel rejected and become avoidant of an unhelpful or an authoritarian approach to care.^25,26,37^

## Innovations for eco-social narrative interventions

The notion of eco-social and structural interventions may seem far removed from the daily work of psychiatrists, and mental health professionals generally. However, when facing traumatised refugees, the trauma narrative testimony and the medicalisation of distress become a gateway to legislative endorsement of refugee status, forcing a narrowed lens. I have outlined why a broader perspective is essential to engage people in an authentic recovery process. There is now interest in more complex trauma, and cognitive–behavioural therapy treatments are recognised to be of value for traumatised adults and children, albeit requiring cultural adaptation of philosophical, technical, theoretical and practical aspects of therapy.^38,39^ Narrative exposure therapy is a promising intervention developed in low-resource settings, and even amid sustained conflict, seeking to process ‘hot’ memories that trigger fear circuits, and render them as stable as ‘cold’ memories.^40^ The approach uses creative methods, such as using flowers and stones to indicate hot and cold memories, and placing them along a piece of string or rope (representing the timeline/age of the person); such approaches show promise with young people and adults, with systematic review evidence showing moderate effect sizes.^41,42^

A review of quasi-experimental and qualitative studies^43^ found the evidence base for interventions in refugees to be generally poor, but recommended that mental health interventions must address immediate needs and concerns before focusing on past traumas, and consider social groups, group therapy and building a social network in conjunction with pharmacotherapy and individual counselling. The ordering of interventions should be driven by the narrative emphasis given by the patient for immediate concerns, and only then can the clinician's judgement of priorities be carefully explained, negotiated and located within the patient's preferred approach to recovery.

Another approach to developing relevant interventions is to engage with communities in intervention development and delivery. Many models of research and community support are adopting community-based participatory processes, applying strength-based interventions. For example, the Refugee Wellbeing Project^44^ includes the following elements/aims: refugee families and undergraduate advocates paired for 6 months; increase ability to navigate new communities; improve access to community resources; enhance meaningful social roles by valuing cultures, experiences and knowledge; reduce social isolation; and increase community responsiveness to refugees. The project intervention includes learning circles to create connections, improve communication and linguistic skills, reduce social injustice, provide empowerment and agency, leading to less anxiety and depression among refugees.^44^

**Fig. 1:**
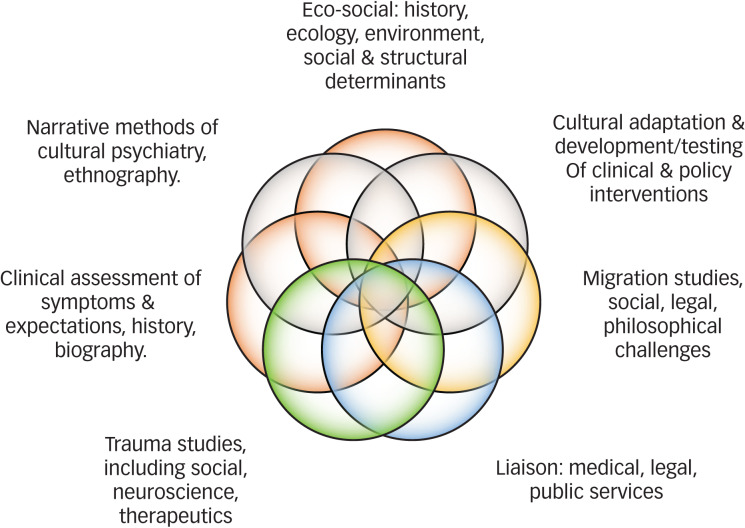
Refugee rose of competencies and capabilities for mental healthcare.

The silos within services fail to recognise the interactions and interfaces of history, the social and cultural world, cognitions and beliefs, morals and political ideologies, and biological vulnerabilities and responses. Indeed, the current health systems are designed to be efficient for care provision and the provider, and rarely to meet the complex experiences of refugees with mental health problems. Narratives in research and clinical practice also reminds all of what is at stake, and emotionally and behaviourally motivate clinicians and policy makers to show courage and address the wider systemic failing of care services.^45,46^ The ‘Refugee Rose’ (Fig. 1) shows the complex influences and bodies of scholarship that need to be aligned for effective assessment, with implications for clinical training, continuing professional development and self-care. Providing assessments and care for refugee populations with multiple adversities and traumas challenges systems of care and our own abilities to empathise, reflect, learn and adapt our interventions and service models. This is a demanding task and vicarious secondary traumatisation of carers and professionals is well established, which can lead to pessimism, depersonalisation, feelings of powerlessness and therapeutic nihilism, especially where there are complex comorbidities such as depression and post-traumatic symptoms, or marked and sustained psychosocial adversity, such that our efforts seem futile until the structural determinants of distress are resolved. In psychodynamic terms, the carer and professional must understand the transference and countertransference (feelings-fears-experience-responses-enactments), and the power of projective identifications, through which the clinician identifies deeply with the unbearable and projected aspects of the patient's internal world. Thus, the clinician must secure appropriate opportunities for review, reflection, supervision and advice. The Cultural Consultation Service ran in East London for 18 months and took 900 referrals. As part of the work of the service, my team established a monthly Cultural Consultation Club, which served exactly the function of giving clinicians and practitioners a creative space and opportunity to discuss aspects of care that were not easily raised in increasingly pressured and resource strained public services. The principles were adopted from the world of cultural psychiatry, using ethnography and thick description, anthropological and social science perspectives, and bringing theory and frameworks of practice together to expose highly structured and often inflexible care pathways. There are good examples of narrative and person-centred approaches to assessment and intervention that also provide social support and care to the most marginalised. Such interventions necessarily must work at the complex intersections of multiple adversities, socioeconomic adversity, and isolation and victimisation.^47,48^ Only through such innovations will we address the complexity of need among those facing historical and contemporary adversity while continuing to live in positions of marginalisation and exclusion. Unpacking the institutional and societal drivers of such intersectional inequalities, including discrimination and racism, have been exposed by the recent COVID0-19 pandemic,^49,50^ showing that social and health systems reforms are needed. Ultimately, political commitment and the organised efforts of society can provide innovation and sustainable solutions, albeit research and practice must continue to be grounded in broad-based evidence and counter institutional and historical forces that sustain hostile and unevidenced policies and practices.
