# A Refugee Rose of competencies and capabilities for mental healthcare of refugees – CORRIGENDUM

**DOI:** 10.1192/bjo.2022.39

**Published:** 2022-03-09

**Authors:** Kamaldeep Bhui

**Keywords:** Refugee, mental health, culture, ethnography, eco-social

In the original publication,^1^ an error was made in the introduction. The first sentence, “The United Nations Humanitarian Commission for Refugees (UNHCR)” should say “United Nations High Commissioner for Refugees (UNCHR)”.

The author apologises for this error.
